# Bayesian estimation of potential outcomes for mediation analysis of racial disparity for infant mortality

**DOI:** 10.21203/rs.3.rs-2874047/v1

**Published:** 2023-05-31

**Authors:** J.A. Thompson

**Affiliations:** College of Veterinary Medicine and Biomedical Science, Texas A&M University, College Station, TX, 77843-4475, USA

**Keywords:** Bayesian, mediation modeling, low birth weight, infant mortality, smoking, teenage maternity

## Abstract

**Background:**

There is a need for novel methods to determine preventable causes of racial health disparities. This need has been met with the development of improved methods for mediation modeling. Current mediational analysis methods call for an evaluation of statistical interaction or effect modification between the investigated cause and mediator. For racial disparity, this approach facilitates the estimation of racially specific risks for infant mortality. However, current methods for evaluating multiple interacting mediators are inadequate. The first objective of the study was to compare Bayesian estimation of potential outcomes to other approaches to mediation analysis that included interaction. The second objective was to evaluate three potentially interacting mediators of racial disparity for infant mortality by modeling the large dataset from the National Natality Database using Bayesian estimation of potential outcomes.

**Methods.:**

A random sample of observations from the 2003 National Natality Database was used to compare the currently promoted methods for mediation modeling. Racial disparity was modeled as a separate function for each of three potential mediators, (i) maternal smoking, (ii) low birth weight and (iii) teenage maternity. As a second objective, direct Bayesian estimation of potential outcomes modeled infant mortality as function of the interactions among the three mediators and race using the full National Natality Database for the years 2016 to 2018.

**Results:**

The counterfactual model was inaccurate in estimating the proportion of racial disparity that was attributable to either maternal smoking or teenage maternity. The counterfactual approach did not accurately estimate the probabilities defined by counterfactual definitions. The error was a result of modeling the excess relative risk instead of the risk probabilities. Bayesian approaches did estimate the probabilities of the counterfactual definitions. Results showed that 73% of the racial disparity for infant mortality was attributed to infants born with low birth weight.

**Conclusions.:**

Bayesian estimation of potential outcomes could evaluate whether proposed public health programs would affect races differently and decisions could include consideration of the causal effect the program may have on racial disparity. The large contribution of low birth weight to racial disparity for infant mortality should be further investigated to identify preventable factors for low birth weight.

## Background

In United States, compared to white infants, Black infants are twice as likely to die in their first year of life. While the overall rate of infant mortality has been decreasing over several decades, the racial disparity, defined as the difference between races, has increased [1]. Recently, the National Institute on Minority Health and Health Disparities (NIMHD) supported a special issue that focused on addressing the causes of racial health disparities in the United States [2, 3]. The authors, of that special issue, concluded that novel methods are necessary to identify causes whose manipulations could form the bases of preventive care programs [4, 5]. While race, itself cannot be manipulated, it may be possible to identify factors that mediate or cause the racial disparity. Existing clinical recommendations and public health programs could be based on preventative modalities that are more effective for white women and their children, so evaluating the factors that mediate or cause racial disparity is imperative to addressing racial disparity in infant mortality. The scientific approach to identifying the sub-component causes of the racial disparity and to evaluate possible structural racism in health programs is called mediation modeling [6].

Considerable methodological development on mediation analysis within the causal-inference literature has been reported over the last ten years and may provide the novel methods called for by the NIHMD [7]. The advancements include mathematical tools for mediation modeling that are based on theoretical counterfactuals often referred to as alternative outcomes. These changes have provided valid methods of making causal inferences from observational data [8]. Originally, the approach was limited to the evaluation of causal variables only at specific levels of the mediating variable [9]. This reasoning was accepted and advanced by modeling and interpreting the interaction, thereby estimating the causal effect not at one level of mediator but at all levels of the mediator in a modeling approach based on structural equations [7]. Structural Equation Modeling (SEM) estimates parameters from multiple equations and combine these parameters to provide estimations of causal effects. However, estimating the variance of functions utilizing estimates from separate equations continues to be problematic among the frequentist approaches. Bayesian analysis implemented using Markov chain Monte Carlo estimation, accounts for asymmetrical (non-normal) distribution of effects [10]. The Bayesian implementation of the SEM approach that includes interaction between the cause and the mediator provides promise for implementing and interpreting single cause and single mediator models. An alternative direct Bayesian approach that does not include counterfactual considerations has long been proposed [11–13]. Under a model with interaction between the cause of interest and the potential mediators, and following the Bayesian assumption of exchangeability, the risk for all combinations of cause and mediation can be estimated directly and these constitute the full set of potential outcomes. Furthermore, extending this approach can estimate potential outcomes from combinations of multiple mediators. In a Bayesian approach, the potential outcomes are estimated for every member of the population for all potential levels of the interaction among mediators.

This study’s objective was to evaluate three potential mediators of racial disparity for infant mortality, including maternal smoking, low birthweight, and teenage maternity. The study implements direct Bayesian estimation of potential outcomes using aggregated counts of observations for each level of race and potential mediators. To illustrate the novel approach, results from direct Bayesian estimation of potential outcomes will be compared to the mediation approach based on a counterfactual model and a Bayesian SEM approach with both models including the estimation of interaction effects. Models will be compared using a smaller database with 100,000 random observations taken from 2003 national U.S. natality database birth records. The direct Bayesian model will then be applied to approximately 11 million observations from the U.S. natality database for the years 2016 to 2018.

## Methods

The source of data was the National Vital Statistics System who provide the data online (https://www.cdc.gov/nchs/nvss/births.htm). In the United States, State laws require birth certificates to be completed for all births, and federal law mandates national collection and publication of births and other vital statistics data. The National Vital Statistics System, the federal compilation of these data, is the result of the cooperation between the National Center for Health Statistics (NCHS) and the states to provide access to statistical information from birth certificates.

The first objective of the study was to compare direct Bayesian estimation of potential outcomes to counterfactual modeling and Bayesian SEM using a manageably sized dataset. This dataset was a random sample of 100,000 births from 2003. These data are provided by SAS^®^ to illustrate mediation modeling [14]. The second objective was to repeat direct Bayesian estimation of outcome potentials for the approximately 11 million singleton births occurring in the U.S. during the three years 2016 to 2018. Variable coding was similar for both databases. Infant mortality was death before one year of age. Maternal race was the factor of interest (X=race) and was coded as either Black or other. Throughout the manuscript, the risk of race is referred to as racial disparity. The potential mediators included maternal smoking (whether the mother reported smoking during pregnancy), low birth weight (LBW; defined as < 2.5 kg) and teenage maternity (defined as maternal age less than 20 years).

For the first objective, four models were implemented. Models 1 and 2 used binary coding for both race (0 = white, 1 = Black) and mediator (factor absent = 0, factor present = 1).

For all models, proportion attributable (PA) to the mediator(s) was estimated as:

(Eq. 1) PA=(TE-CDE)/TE

Model 1 was a counterfactual method known as a four-way decomposition [7]. This approach relies on estimations from SEM that includes the all-factor regression equation:

(Eq. 2) $$\text{Logit}\left(\text{Y}\left[\text{i}\right]\right)=\;\text{alpha}\;+{\;\text{cprime*race}}_{\text{i}}+{\text{b*M}}_{\text{i}}+{\text{h*race}}_{\text{i}}{\text{*M}}_{\text{i}}$$

The approach uses the regression parameters algebraically to partition components of risk as a portion of the total risk, estimated as the portion of relative risk that exceeds 1.

Model 2 was a Bayesian Structural Equation Model [10]. For i=100,000 observations, $Y_{i}$ was distributed as Poisson with rate parameter $u_{i}$ .


(Eq. 3) $$Y_{i}∼Poisson\left(u_{i}\right)$$

The logit of $u_{i}$ entered into two structural equations.


(Eq. 4) $$\text{Logit}\left({\text{u}}_{\text{i}}\right)<-\;\mathrm{alpha.c}+{\text{c*race}}_{\text{i}}$$

(Eq. 5) $$\mathrm{Logit}\left({\text{u}}_{\text{i}}\right)<-\;\mathrm{alpha}+\text{cprime*}{\mathrm{race}}_{\text{i}}+{\text{b*M}}_{\text{i}}+{\text{h*race}}_{\text{i}}*{\text{M}}_{\text{i}}$$

Total effect (TE) and controlled direct effect (CDE) were estimated using the reverse transform of the log odds of c and cprime, respectively.


(Eq. 6) TE<−exp(alpha.c+c)/(exp(alpha.c+c)+1)−exp(alpha.c)/(exp(alpha.c)+1)

(Eq. 7) CDE<−exp(alpha+cprime)/(exp(alpha+cprime)+1)−exp(alpha)/(exp(alpha)+1)

Models 3 and 4 estimated the rates for potential outcomes under Bayesian implementations. Race (X) and mediators (M) were coded as categorical variables. Race was category = 1 for white and category = 2 for Black and mediators were coded category = 1 for factor absent and category = 2 for factor present.

In model 3, for i=100,000 observations, $Y_{i}$ (coded as 0 or 1) was modeled as Bernoulli with an unknown rate parameter $p_{Xi,Mi}$ .


(Eq. 8) $$Y_{i}∼Bernoulli\left(p\left[X_{i},M_{i}\right]\right)$$

The four rate parameters are the potential outcomes had uniform(0,1) priors.


(Eq. 9) p[1:2,1:2]∼Uniform(0,1)

The total effect (TE) was the rate of Y for race = Black and mediator value = missing minus the rate of Y for race = white and mediator value = missing.


(Eq. 10) TE<-p[2,NA]-p[1,NA]

The controlled direct effect CDE was estimated as the rate of Y for race = Black and mediator = 1 minus the rate of Y for race = white and mediator = 1.


(Eq. 11) CDE<-p[2,1]-p[1,1]

For model 4, the rates for potential outcomes were estimated using direct Bayesian estimation on tabulated data and a Binomial prior. Data were aggregated into four rows with each row identified by unique values for X and M. For rows i=1 to 4,, the occurrences of infant mortality were counted as $r_{i}$ and the number of births as $n_{i}$. The count of deaths $r_{i}$ was modeled as Binomial with a rate parameter $p\left[X_{i},M_{i}\right]$ and count of births $n_{i}$.


(Eq. 12) $$r_{i}∼Binomial\left(p\left[X_{i},M_{i}\right],n_{i}\right)$$

The four rate parameters are the potential outcomes and were given uniform(0,1) priors.


(Eq. 13) p[1:2,1:2]∼Uniform(0,1)

The total effect (TE) was the rate of Y for race = Black and mediator value = missing minus the rate of Y for race = white and mediator value = missing.


(Eq. 14) TE<-p[2,NA]-p[1,NA]

The controlled direct effect (CDE) was estimated as the rate of Y for race = Black and mediator = 1 minus the rate of Y for race = white and mediator = 1.


(Eq. 15) CDE<-p[2,1]-p[1,1]

For the second objective, Model 5 estimated rates for potential outcomes using direct Bayesian estimation on tabulated data and a Binomial prior. Data were aggregated into 16 rows with each row identified by unique values for X and three mediators (M1,M2,M3). For i in 16 rows of data, the occurrences of infant mortality were counted as $r_{i}$ and the number of births as $n_{i}$. The count of deaths $r_{i}$ was modeled as Binomial with a rate parameter $p\left[X_{i},\;M1_{i},\;M2_{i},\;M3_{i}\right]$ and count of births $n_{i}$ .


(Eq. 16) $$r_{i}∼Binomial\left(p\left[X_{i},{M1}_{i},{M2}_{i},{M3}_{i}\right],n_{i}\right)$$

The i=16 rate parameters are the potential outcomes and were given Uniform(0,1) priors.


(Eq. 17) p[1:2,1:2,1:2,1:2]∼Uniform

The standard counterfactual definition was used as TE:

(Eq. 18) Prob⁡(Y)|X=2-Prob⁡(Y)|X=1

The TE was taken from the model as:

(Eq. 19) p[2,NA,NA,NA]-p[1,NA,NA,NA]

To estimate controlled direct effect (CDE), the counterfactual definitions and model estimators used are listed in Table 1:

For all Bayesian modeling, estimation was performed using Multibugs 1.0 [15]. A burn-in of 5,000 iterations was discarded and the next 10,000 iterations were collected for posterior distributions. Convergence was determined by monitoring chains with disparate starting values. Reported results are the mean and 95% credibility interval which were the 2.5 and 97.5 percentiles taken directly from the posterior distribution. To evaluate skewness of posterior distributions, the sample mean and median were compared.

## Results

For objective 1, the models produced very similar results for the mediation effect of low birth weight including both the mean effect and the variability of the effect. For the counterfactual model, the estimates for PA for maternal smoking and teenage maternity were different than for the other models (Table 2). The confidence interval values for the counterfactual model were identical to a single decimal for both the Delta and bootstrap methods.

Parameters from regression modeling showed that estimates for both the counterfactual model (Eq. 2) and the Bayesian SEM model (Eq. 5) were similar (Additional file 1).

Births from the U.S. natality database for the three years, 2016 to 2018 were followed for one year, including until December 31, 2019, for the incidence of infant mortality. The records identified 11,226,394 singleton births for analyses.

The Deviance Information Criterion (DIC) was estimated to evaluate goodness of fit among the models. The best fitting model included all interactions among race and the three mediators. The four-way interaction model was shown to be far superior to reduced models (Table 3).

The modeling results are based on the 16 outcome probabilities, defined by combinations of race, smoking, low birth weight and teenage maternity (Table 4).

The public health benefit is the population’s reduction in racial disparity for mediating (completely preventing) one or more of the alterable risk factors. The mediated proportion estimated using the Bayesian model showed virtually no mediation of racial disparity for maternal smoking and teenage maternity and maternal smoking combined with teenage maternity. The proportion mediated by low birth weight was 73.0 (71.0, 74.9) and the proportion mediated by low birth weight combined with maternal smoking and by low birth weight combined with teenage maternity was slightly higher but very similar to the mediation of low birth weight, alone (Table 5). Maternal smoking and teenage maternity had little evidence to support their cause of racial disparity in infant mortality. Low birth weight was estimated to cause over 73% of the population effect known as racial disparity for infant mortality.

## Discussion

Counterfactual modeling was developed and promoted to estimate causal effects. The causal effect evaluated in this study is racial disparity defined as the effect of race on infant mortality. A patient’s race cannot be altered but, the current study aims to evaluate whether three known causes of infant mortality cause all or part of the racial disparity. Counterfactual or potential outcome modeling has addressed the limitation that a modeler cannot know a patient’s alternative outcome that would have occurred if the facts had changed and the mediator under consideration had been different. In Bayesian modeling, the directly estimated potential outcome applies to all individuals in the strata defined by X (race) and M (mediators). A patient’s alternative outcome, should the mediator be changed, is estimated as the disease rate in the new stratum defined by X (race) and the new mediator (M). The Bayesian assumption of exchangeability replaces the four conditions necessary to justify the counterfactual approach [7].

Proponents of counterfactual modeling propose definitions of the target probabilities [7]. For example, the proportion of racial disparity attributable to maternal smoking would need estimates for the probability of racial disparity, ignoring the mediator which is called the TE as well as the effect controlling for maternal smoking equals “no.” Total effect for racial disparity is a probability equal to: Probability (infant mortality = yes if race = Black) – probability (infant mortality = yes if race = white). Controlled direct effects are estimable as a probability. For example, the racial disparity for considering the non-smoking mothers would be the CDE for maternal smoking which was: Probability (infant mortality = yes, if race = Black and maternal smoking = no) minus probability (infant mortality = yes, if race = white and maternal smoking = no). The attributable proportion is a probability defined as (TE-CDE)/TE. Based on these defined probabilities, the evaluated counterfactual model estimated the proportion of racial disparity attributable to low birth weight accurately but was inaccurate for predicting the proportion of racial disparity attributable to maternal smoking or teenage maternity. The error can be attributed to the partitioning of excess relative risk, defined as the amount that the relative risk for racial disparity was greater than one. The counterfactual model estimated the excess relative risk for both maternal smoking and teenage maternity as negative and statistically significant near the P=0.05 critical value. These results, if accurate, would support the conclusion that successful mediation of teenage maternity and maternal smoking would cause a statistically significant increase in racial disparity. The Bayesian results directly estimate the defined counterfactual probabilities which also showed a negative mean effect for PA, but the estimates had much wider confidence intervals that included zero. Direct Bayesian estimation was very similar whether data were individual Bernoulli observations or tabulated in Binomial format. Comparing the two data formats, mediation estimates were similar, they varied by a small amount that could be attributed to Monte Carlo error and slightly different vague priors. In addition to the direct Bayesian estimation approach, the Bayesian SEM approach also produced estimates that matched the counterfactual probabilities. Both the Bayesian SEM and counterfactual approaches rely on regression equations that estimate regression parameters for an intercept, the cause (X), the mediation (M) and the interaction (X*M). The two methods differ in implementation (see equations 2 and 5, in Methods) but in this study the regression parameters were nearly identical. This showed that the observed error in the counterfactual mediational estimates can be attributed to how the regression estimates were used to model a difference in risk ratios. The resulting counterfactual estimates did not match the definition of the probabilities they were intended to.

**Figure 1:**
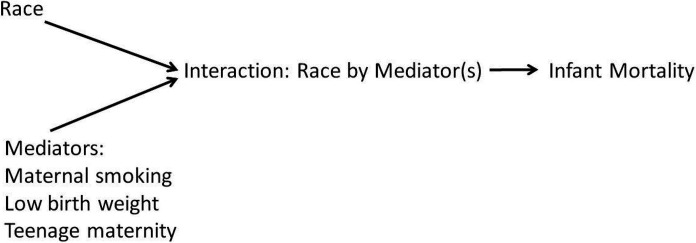
The DAG used in the current study.

Mediation of causal effects when the cause and mediator interact should be consistent with a structural causal model and must be representable by a directed acyclic graph (DAG) [16, 17]. Faced with the quandary of fitting interaction into a DAG, the investigators adopted a prior belief consistent with a proposed causal structure. The investigators’ prior belief was that the interaction represented a unique node with estimable risks. Furthermore, on condition that the interaction risk is separable and representable by a node, race cannot cause the mediator because when race does influence incidence of the mediator, race is causing the interaction (Fig. 1).

With race (X) and mediator (M) binary, the interaction race by mediator has four levels. The DAG shows that M and X are confounded by a common downstream variable (the interaction X*M) and to estimate the full set of causal effects, the fully conditional estimates are needed (equations 2 and 5 in Methods). For the direct Bayesian aproaches, estimation of the probability of Y(pY) at each level of interaction provides the full set of potential outcomes (pY∣X,M). Thus, the proposed DAG is consistent with all of the modiling approaches compared in the current study and the DAG is consistent with counterfactual definitions. The investigators encourage further investigations incorporating alternative prior beleifs that are representable by alternative DAGs.

With direct estimation of potential outcomes, the single cause and single mediatior DAG can readily be expanded to multiple mediators and single cause with both interaction and mediation. The DAG used to guide the second objective of the current study proposed a 16 level interaction node. This prior belief is also consistent with estimation equations and counterfactual definitions defined previously for multiple mediators interacting among themselves and with a cause [18]. The 16 potential outcomes are defined as the probabilty of infant mortality conditional upon race and mediatiors (pY∣X,M1,M2,M3) and, adopting the Bayesian condition of exchangebility, these potential outcomes apply to all individuals in the stratum defined by X,M1,M2 and M3. In the approach, the assumption is singular and easily testible. To extend the SEM modeling to include multiple interacting mediators, the system of equations would become complex with estimation of effects for all interactions which, in the current study would have included six, two-way interactions, four, three-way interactions and one four-way interaction. Further expansion of modeling in this direction is possible but will be difficult to implement and interpret because of the complexity of combining the effects [18].

The assumption of exchangebility when data are tabulated is essentially that there are no other variables identifying the probability of infant mortality within any spsecific stratum. This can be tested by entering the variable into the interaction and evaluating the DIC. When the variable enters as an interaction and the model fit is superior, the conclusion is that the previous data were not conditionally exchangeable. Bayesian proponents often claim that the condition of exchangeablity is more relaxed than the comparable frequentist assumpions. In the proposed application, the assumption is not less restrictive. The results from this study showed that the best fitting model included interactions among all three mediators which confirms that the data for three single mediator interactions with race were not exchangeable. Further, it can be expected that more variables can be entered thus nullifying the three mediator model. The assumption of exchangeablity is not more relaxed – it is merely simpler to move forward in the modeling process. The modeling showed that the three mediator model was superior to the single mediator model with LBW mediating racial disparity but the superior models do little to change the estimate of the proportion of racial disparity attributable to LBW. The conclusion is that approximately 75% of the racial disparity for infant mortality was due to low birth weight but the proportion varied a small amount with maternal smoking and teenage maternity.

Investigators committed to a Bayesian approach should recognize that the probability is near certain that at least one cause of missing infant mortality is missing from at least one stratum and these missing variables provide a modeling limitation. Under the modeling proposed here, these missing variables should be considered potential effect modifiers of racial disparity rather than confounders contributing to bias. For example, the pre-submission reviewers rejected the results, presented here, because low birth weight could have been confounded with premature delivery. Certainly the two factors will interact because low birth weight is either caused by intrauterine growth restriction or preterm birth. Intrauterine growth restriction can cause preterm birth and subsequent infant mortality and preterm birth can cause low birth weight and subsequent infant mortality. Thus, low birth weight and preterm birth will interact in causing infant mortality and the current model should be expanded to include preterm birth. Following the proposed methodology, not knowing the causal ordering between LBW and preterm birth is no longer limiting the mediation analysis. The proportion of racial disparity attributable to either or both can be estimated. The overall average effect of LBW on racial disparity will remain the same but the extent of racial disparity can be expected to vary along with gestation length.

As more causes of racial disparity are identified, the complexity of models will become a limitation. Evaluating DIC as more variables are evaluated will show that each additional variable adds an increasing number of effective parameters. Frequentists will recognize this as a similarity to increasing degrees of freedom for interaction models. In the reported modeling, race plus intercept has two effective parameters. Adding the first mediator increased effective parameters to four and adding the second mediator increased the effective parameters to eight and the third mediator to 16 effective parameters. The current study had over 11 million observations, but most studies will model many fewer data. There are at least two reasonable responses. The first is to reduce the number of levels of interaction. For example, in a hypothetical model, it is possible that a single combination of three mediators could be the cause of 100% racial disparity. Such a model could be reduced to two effective parameters (intercept and a single combination of race, M1,M2 and M3). To implement a reduced model, a single variable for each combination of the mediators can be created and tested for entry using the DIC. As a second strategy, as models become more complex, it is a reasonable Bayesian approach to use more informative prior values. The current study used priors for infant mortality that were uniform (0,1). This prior was selected to be minimally informative but there is no such prior as one that is totally uninformative. The prior used in the current study gave the same prior probability for a mortality rate of 1 (each child died in the stratum) to a much more realistic probability of 0.001 (chance of child dying who was in the stratum of 1 in 1000). Bayesian analysts should be more comfortable with a gamma prior with probability mass closer to zero than one.

The racial disparity for infant mortality in the U.S. from three years of observations was largely mediated by low birth weight. The mediation model predicts that if low birth weight could be prevented, nearly 75% of the racial disparity for infant mortality would be alleviated. Besides different rates between the two race groups for both prevalence of mediators and race-specific risks there also exists a difference in acceptance of public health programs. A legacy of racial discrimination in medical research and the health care system has been linked to a low level of trust in medical research and medical care among African Americans [19]. This mistrust is associated with perceived discrimination and addressing this perception is one of the key elements of addressing racial health disparities [20]. To address this perception, direct Bayesian estimation of the PA of mediators toward racial disparity could be used to ensure racial fairness.

## Conclusions

The causes of racial disparity for infant mortality need more research. A Bayesian mediation modeling approach has shown remarkable potential for identifying the mediating causes.

## Figures and Tables

**Table 1:** The counterfactual definition for CDE defines the probability of infant mortality P(Y) conditional upon specific values for race (X) and mediators M1 (smoking), M2 (LBW) and M3 (teenage maternity). The model estimator is the value from the model that estimates the CDE. A value of NA means the indicator value was treated as missing.

Mediator	Counterfactual definition of CDE	Model estimator of CDE
M1	P(Y)|X = 2,M1 =1-P(Y)|X = 1,M1 =1	p[2,1,NA,NA]-p[1,1,NA,NA]
M2	P(Y) |X = 2,M2 = 1- P(Y) |X = 1 ,M2 = 1	p[2,NA,1,NA]-p[1,NA,1,NA]
M3	P(Y)|X = 2,M3 = 1-P(Y)|X = 1,M3 = 1	p[2,NA,NA,1]-p[1,NA,NA,1]
M1,M2	P(Y)|X = 2,M1 =1,M2 = 1-P(Y)|X = 1,M1 =1,M2 = 1	p[2,1,1,NA]-p[1,1,1 ,NA]
M1,M3	P(Y)|X = 2,M1 =1,M3 = 1-P(Y)|X = 1,M1 =1,M3 = 1	p[2,1,NA,1]-p[1,1 ,NA,1 ]
M2,M3	P(Y)|X = 2,M2 = 1,M3 = 1-P(Y)|X = 1,M1 = 1,M3 = 1	p[2,NA,1,1]-p[1 ,NA,1,1 ]
M1,M2,M3	P(Y)|X = 2,M1 = 1,M2 = 1,M3 = 1-P(Y)|X = 1,M1 = 1,M2 = 1,M3 = 1	p[2,1,1,1]-p[1,1,1,1]

**Table 2:** Comparison of models for estimates of the proportion attributed to single mediating factors. Values given are the effect means and the 95% confidence intervals (often called credible intervals in Bayesian modeling).

	Proportion of effect mediated			
Mediating cause	Counterfactual	Bayesian SEM	Direct Bayesian (Bernoulli)	Direct Bayesian (Binomial)
Maternal smoking	−0.12 (−0.24, 0.01)	−0.14 (−0.70, 0.27)	−0.14 (−0.72, 0.27)	−0.14 (−0.71,0.27)
Low birth weight	0.90 (0.76, 1.04)	0.89 (0.70, 1.04)	0.88 (0.68, 1.03)	0.88 (0.69, 1.03)
Teenage maternity	−0.13 (−0.26, 0.00)	−0.16 (−0.74.5, 0.27)	−0.16 (−0.75, 0.26)	−0.16 (−0.74, 0.26)

**Table 3:** Model fit parameters. Models are listing in order (top to bottom) of improving fit (lower Deviance Information Criterion indicates better fit).

Model	Deviance Information Criterion	Effective parameters
Intercept only	127700	1
Race with no interactions	121400	2
Race interacting with teenage maternity	120700	4
Race interacting with smoking	118300	4
Race interacting with smoking and teenage maternity	117700	8
Race interacting with low birth weight	3023	4
Race interacting with low birth weight and teenage maternity	2621	8
Race interacting with smoking and low birth weight	562	8
Race interacting with smoking, low birth weight and teenage maternity	171	16

**Table 4:** Potential Outcomes. The outcome is the estimate of the probability of an infant’s mortality, based on the maternal factors. In Bayesian analyses, potential outcome (outcome for alternative set of factors) is directly estimable as the difference of risk between the two risk estimates.

Presence of maternal factors (Yes/No)				Probability of Mortality	
Maternal smoking	Low birth weight		Teenage maternity	White infant	Black infant
No	No	No		0.0014 (0.0014, 0.0015)	0.0027 (0.0026, 0.0028)
No	No	Yes		0.0027 (0.0026, 0.0029)	0.0037 (0.0034, 0.0041)
No	Yes	No		0.045 (0.044, 0.045)	0.057 (0.056, 0.058)
No	Yes	Yes		0.052 (0.050, 0.055)	0.056 (0.052, 0.059)
Yes	No	No		0.0044 (0.0042, 0.0046)	0.0071 (0.0065, 0.0077)
Yes	No	Yes		0.0054 (0.0047, 0.0061)	0.0069 (0.0045, 0.0097)
Yes	Yes	No		0.039 (0.038, 0.041)	0.051 (0.048, 0.055)
Yes	Yes	Yes		0.045 (0.039, 0.051)	0.056 (0.041, 0.073)

**Table 5:** Estimates of the proportion of racial disparity attributable to smoking, low birth weight, teenage maternity, and combinations of these mediators.

Mediating factor(s)	Proportion of Racial Disparity Attributed to the mediating factor(s)
Smoking	1.1 (−3.0, 5.2)
Low birth weight	73.0 (71.0, 74.9)
Teenage maternity	−0.2 (−4.4, 4.0)
Smoking, low birth weight	73.4 (71.5, 75.3)
Smoking, teenage maternity	1.6 (−2.7, 5.6)
Low birth weight, teenage maternity	73.1 (71.0, 75.1)
Smoking, low birth weight, teenage maternity	73.9 (71.8, 75.8)

## Data Availability

The data for objective 1 are provided in additional file 2. The data for objective 2 are provided in additional file 3. The statistical codes for all models are available from the corresponding author on reasonable request.

## Abbreviations

CDE: controlled direct effect
DIC: Deviance Information Criterion
LBW: low birth weight
M: the mediational factor of interest (M1, M2 or M3)
M1: smoking (yes or no)
M2: low birth weight (yes or no)
M3: teenage maternity (yes or no)
NCHS: National Center for Health Statistics
NIMHD: National Institute on Minority Health and Health Disparities
PA: percent attributable
pY: probability of Y (infant mortality)
SEM: SEM
TE: total effect
X: the factor of interest for mediational analysis (race)
